# Association of Oral Health Assessment Tool Findings With Periodontal Screening Outcomes in Adults With Diabetes

**DOI:** 10.1002/cre2.70465

**Published:** 2026-09-19

**Authors:** Grace Wong, Sarah Glastras, Sarah Brennan, Marija Saponja, Anna Cheng, Jami Walk, Kyle Cheng, Wenpeng You

**Affiliations:** ^1^ Oral Health Services, Primary and Community Health, Northern Sydney Local Health District, NSW Health New South Wales Australia; ^2^ Sydney Dental School, Faculty of Medicine and Health the University of Sydney New South Wales Australia; ^3^ School of Nursing and Midwifery Western Sydney University New South Wales Australia; ^4^ Department of Diabetes, Endocrinology and Metabolism, Royal North Shore Hospital, Northern Sydney Local Health District NSW Health New South Wales Australia; ^5^ Kolling Institute of Medical Research, Northern Clinical School the University of Sydney New South Wales Australia; ^6^ Aboriginal and Torres Strait Island Health Service, Hornsby Hospital Northern Sydney Local Health District, NSW Health New South Wales Australia; ^7^ Adelaide Medical School the University of Adelaide South Australia Australia; ^8^ Adelaide Nursing School the University of Adelaide South Australia Australia

**Keywords:** diabetes mellitus, Oral Health Assessment Tool, Oral Health Screening: Primary Care, periodontal disease, periodontal screening

## Abstract

**Background:**

Periodontal disease is common among adults with diabetes, yet periodontal screening is rarely incorporated into routine outpatient diabetes care. The Oral Health Assessment Tool (OHAT) is a structured observational tool designed for non‐dental healthcare professionals; however, its relationship with periodontal screening findings remains unclear.

**Objectives:**

To examine the association between OHAT findings and periodontal screening outcomes in adults attending diabetes outpatient clinics.

**Methods:**

This cross‐sectional study included adults with type 1 or type 2 diabetes at a tertiary hospital in Sydney, Australia. Periodontal screening was performed using the Periodontal Screening and Recording (PSR) system, and oral health was assessed using OHAT. Periodontal status was dichotomized as no/mild (PSR 0–2) versus moderate‐to‐severe (PSR ≥ 3). Logistic regression examined associations of OHAT domains and total OHAT burden with periodontal status. Covariate‐adjusted models assessed robustness. Screening performance was evaluated using sensitivity, specificity, and predictive values.

**Results:**

Among 120 participants (mean age 61.5 years, 64.2% with type 2 diabetes), 71.4% demonstrated moderate‐to‐severe periodontal findings. Mean OHAT score was 4.99 (SD 2.02). Change/unhealthy ratings for tongue (OR 3.23, 95% CI 1.30–8.02), gums and oral tissues (OR 4.59, 95% CI 1.78–11.85), and increasing categories of decayed teeth (OR 2.06, 95% CI 1.05–4.05) were associated with moderate‐to‐severe findings. Higher total OHAT score (> 5 vs. ≤ 5) was strongly associated with moderate‐to‐severe periodontal findings (OR 7.00, 95% CI 2.25–21.76), with consistent results. This threshold showed high specificity (87.5%) and positive predictive value (90.9%), but moderate sensitivity (50.0%).

**Conclusion:**

OHAT findings were associated with moderate‐to‐severe periodontal screening findings among adults with diabetes, supporting further evaluation of OHAT as a pragmatic screening and referral aid in settings where formal periodontal assessment is not routinely available. Its performance when administered by non‐dental clinicians requires further investigation.

## 1 Introduction

Diabetes mellitus is one of the most prevalent chronic conditions worldwide and is associated with a range of systemic complications, including periodontal disease (Gregg et al. 2024; Nguyen et al. 2020). Diabetes and periodontal disease exhibit a well‐established bidirectional relationship (Stöhr et al. 2021), whereby chronic hyperglycemia promotes exaggerated inflammatory responses and impaired wound healing within periodontal tissues, while periodontal inflammation may contribute to systemic inflammatory burden and adversely influence glycemic control (Preshaw et al. 2012; Ranbhise et al. 2025). Epidemiological and clinical studies have consistently demonstrated associations between periodontal disease severity and poorer metabolic outcomes in individuals with diabetes (Graves et al. 2026; Genco et al. 2020; Santonocito et al. 2022). However, translation of this evidence into routine periodontal assessment within diabetes care remains limited (Dale et al. 2014; Mamun and Awual 2025).

Structured periodontal screening is not routinely incorporated into many diabetes outpatient settings (Pumerantz et al. 2017). The Periodontal Screening and Recording (PSR) system is widely used in dental practice to identify periodontal pocketing requiring further clinical evaluation (Landry and Jean 2002). However, PSR requires periodontal probing performed by trained oral health professionals and is not feasible in most non‐dental clinical environments, including diabetes clinics (Prasad et al. 2019). As a result, individuals with diabetes at elevated risk of periodontal disease may not be systematically identified unless they access dental services independently.

The Oral Health Assessment Tool (OHAT) is a structured, non‐invasive assessment designed for use by non‐dental healthcare professionals. Adapted from the Brief Oral Health Status Examination (Kayser‐Jones et al. 1995), OHAT evaluates eight observable domains—lips, tongue, gums and oral tissues, saliva, natural teeth, dentures, oral cleanliness, and dental pain using standardized criteria for healthy, change, or unhealthy findings (Chalmers et al. 2005). OHAT has demonstrated acceptable reliability and feasibility and has been widely adopted in inpatient and aged care settings (Everaars et al. 2020; Thapa et al. 2021; Chu et al. 2024). However, its relationship with periodontal screening findings in adults with diabetes has not been well characterized.

Although OHAT does not directly assess periodontal pocketing or attachment loss, several domains capture observable changes potentially relevant to periodontal status, including gingival appearance, oral cleanliness, and natural teeth condition. It remains unclear whether these observable indicators correspond to periodontal screening findings in adults with diabetes. Clarifying this relationship is important to examine whether structured observational assessment may assist in identifying individuals who warrant further periodontal evaluation.

Accordingly, this study examined the association of OHAT findings with periodontal screening results among adults attending diabetes outpatient clinics, using PSR as the reference standard. It was hypothesized that higher total OHAT burden and increasing severity of domain‐level changes would be associated with greater odds of moderate‐to‐severe periodontal screening findings.

## 2 Methods

### 2.1 Study Design and Setting

This cross‐sectional study was conducted in the diabetes, endocrinology, and podiatry outpatient clinics at Royal North Shore Hospital in the Northern Sydney Local Health District and was approved by the Northern Sydney Local Health District Human Research Ethics Committee (2023/02593).

Participants underwent oral health assessment using the Oral Health Assessment Tool (OHAT) and concurrent periodontal screening using the Periodontal Screening and Recording (PSR) system during a single clinic visit.

### 2.2 Participant Recruitment

Adults aged 18 years or older with a confirmed diagnosis of type 1 or type 2 diabetes attending routine outpatient clinics during the study period were eligible for inclusion. Eligibility was based solely on age and diabetes diagnosis to minimize selection bias. Individuals with secondary or pancreatogenic diabetes were excluded due to differences in underlying pathophysiology, inflammatory mechanisms, and clinical management compared with type 1 and type 2 diabetes.

Participants were recruited using convenience sampling during routine appointments between November 2024 and June 2025. Consecutive eligible patients attending clinic sessions were approached by members of the multidisciplinary care team using a standardized script. Patients were not selected based on oral health concerns, perceived oral hygiene, or visible oral conditions.

All participants provided written informed consent. Following consent, participants completed the structured study questionnaire and underwent the oral health assessments during the same outpatient clinic visit; no separate study appointment was required. The number of eligible patients approached who declined participation was not systematically recorded.

Study procedures adhered to the National Statement on Ethical Conduct in Human Research (NHMRC, Australia).

### 2.3 Data Collection Procedures

Following consent, participants completed a structured questionnaire capturing demographic characteristics, diabetes history (type and duration), medication use, diabetes‐related complications, oral hygiene practices, dental attendance patterns, oral health symptoms, perceived barriers to dental care, and awareness of the diabetes–oral health relationship. Questionnaire data were used to characterize the study sample.

Immediately following completion of the questionnaire, participants then underwent a standardized oral health assessment in the outpatient clinic during the same visit. The assessment was conducted by a qualified oral health professional using a portable dental chair, mouth mirror, a color‐coded periodontal probe (CP12), and a head‐mounted light. Periodontal screening was performed first using the Periodontal Screening and Recording (PSR) system according to the standard sextant‐based protocol. A PSR score ≥ 3 indicates periodontal pocketing requiring further clinical evaluation (Landry and Jean 2002).

Following the PSR assessment, oral health status was assessed using the Oral Health Assessment Tool (OHAT), which evaluates eight oral health domains (lips, tongue, gums and oral tissues, saliva, natural teeth, dentures, oral cleanliness and dental pain). Each domain is scored as healthy (0), change (1), or unhealthy (2), yielding a total OHAT score ranging from 0 to 16. Higher scores indicate greater severity and accumulation of observed oral health changes (Chalmers et al. 2005).

All OHAT and PSR assessments were performed by a single experienced oral health clinician; however, inter‐rater reliability was not assessed. In this study, PSR was used as a screening reference standard to determine whether OHAT domains and total OHAT burden were associated with moderate‐to‐severe periodontal findings.

Biomedical data, including the most recent HbA1c (NGSP%), total cholesterol, LDL cholesterol, and serum creatinine values, were extracted from electronic medical records with participant consent. These variables were used to describe metabolic status and to assess the robustness of observed associations in exploratory analyses.

Behavioral variables were not included in regression analyses to maintain focus on OHAT as a clinically observable screening measure and to minimize overadjustment, as behavioral factors may lie on the causal pathway between oral health status and periodontal findings.

### 2.4 Outcome Definition

For primary analyses, periodontal status was dichotomized as no or mild periodontal screening findings (PSR 0–2 across all sextants) versus moderate‐to‐severe periodontal screening findings (PSR ≥ 3 in ≥ 1 sextant). PSR was treated as a screening reference standard rather than a diagnostic measure of periodontal disease severity.

### 2.5 Statistical Analysis

Descriptive statistics were used to summarize participant characteristics, OHAT findings, and periodontal screening outcomes. Continuous variables are presented as means and standard deviations or medians and interquartile ranges, as appropriate. Categorical variables are presented as frequencies and percentages.

Associations between individual OHAT domains and PSR–defined moderate‐to‐severe periodontal findings were examined using binary logistic regression. Each OHAT domain was dichotomized as healthy versus change/unhealthy. The decayed teeth domain was analyzed as an ordinal variable reflecting increasing caries burden. Total OHAT burden was analyzed using a binary variable (> 5 vs. ≤ 5), using a pre‐defined threshold to represent higher oral health burden. Odds ratios (ORs) with 95% confidence intervals (CIs) were calculated.

To assess the robustness of observed associations, separate covariate‐adjusted logistic regression models were fitted for each OHAT variable by entering one covariate at a time. Adjustment variables included age, diabetes type, gender, HbA1c, creatinine, total cholesterol, and LDL cholesterol. This approach was used to minimize model instability and overfitting, given the sample size and missing data across some biomedical variables.

Sensitivity, specificity, positive predictive value (PPV), and negative predictive value (NPV) were calculated to evaluate the screening performance of individual OHAT domains and the total OHAT burden against PSR‐defined moderate‐to‐severe periodontal findings.

All analyses were conducted using IBM SPSS Statistics (version 31). Statistical significance was defined as a two‐sided *p*‐value < 0.05.

## 3 Results

### 3.1 Participant Characteristics and Distribution of Oral Health Outcomes (Table 1)

**Table 1:** Participant characteristics, clinical indicators, and oral health status (*N* = 120).

Demographic Characteristics	Category/statistic	n (%) or mean ± SD
Age (years)	Mean ± SD (range)	61.48 ± 17.30 (20.15–88.82)
Gender	Male	65 (54.2)
	Female	55 (45.8)
Aboriginal and/or Torres Strait Islander status	No	5 (4.2)
	Yes	115 (95.8)
Diabetes and clinical characteristics		
Diabetes type	Type 1	43 (35.8)
	Type 2	77 (64.2)
HbA1c NGSP (%)	Mean ± SD (range)	7.77 ± 1.82 (4.00–14.00)
LDL cholesterol (mmol/L)	Mean ± SD (range)	2.20 ± 0.99 (0.30–5.80)
Total cholesterol (mmol/L)	Mean ± SD (range)	4.13 ± 1.13 (1.50–8.00)
Creatinine (mmol/L)	Mean ± SD (range)	103.64 ± 96.07 (45–856)
Periodontal screening		
PSR category	Mild	32 (28.6)
	Moderate	13 (11.6)c
	Severe	67 (59.8)
OHAT findings		
OHAT—Lips	Healthy	16 (13.3)
	Changes	103 (85.8)
	Unhealthy	1 (0.8)
OHAT—Tongue	Healthy	27 (22.5)
	Changes	92 (76.7)
	Unhealthy	1 (0.8)
OHAT—Gums and oral tissues	Healthy	63 (52.5)
	Changes	52 (43.3)
	Unhealthy	5 (4.2)
OHAT—Saliva	Healthy	38 (31.7)
	Changes	77 (64.2)
	Unhealthy	5 (4.2)
OHAT—Natural teeth	Yes	110 (92.4)
	No	9 (7.6)
OHAT—Decayed teeth†	None	52 (47.3)
	1–3 teeth	44 (40.0)
	≥ 4 teeth	14 (12.7)
OHAT—Dentures	Yes	26 (21.7)
	No	94(78.3)
OHAT—Type of denture‡	Full/Full	7 (26.9)
	Full/Partial	2 (7.7)
	Partial/Full	None identified
	Partial/Partial	7 (26.9)
	Partial/None	4 (15.4)
	Full/None	1 (3.8)
	None/Partial	4 (15.4)
	None/Full	1 (3.8)
OHAT—Denture condition	Good	14 (53.8)
	Fair	6 (23.1)
	Poor	6 (23.1)
OHAT—Oral cleanliness	Clean	18 (15.0)
	Moderate	63 (52.5)
	Poor	39 (32.5)
OHAT—Dental pain	No pain	106 (88.3)
	Occasional	8 (6.7)
	Frequent	6 (5.0)

*Note:* Data are presented as *n* (%) or mean ± SD (range). Percentages are based on valid cases. Higher OHAT scores indicate poorer oral health.

Abbreviations: OHAT, Oral Health Assessment Tool; PSR, Periodontal Screening and Recording.

† Decayed teeth are reported for participants with natural teeth (*n* = 110).

^‡^ Denture‐related variables are reported for denture wearers only (*n* = 26).

A total of 120 participants were included. The mean age was 61.5 years (SD 17.3; range 20.1–88.8). Type 2 diabetes was more prevalent than type 1 (64.2% vs. 35.8%), and 54.2% of participants were male. Most participants identified as non‐Indigenous (95.8%).

Mean HbA1c was 7.8% (SD 1.8), LDL cholesterol 2.20 mmol/L (SD 0.99), total cholesterol 4.13 mmol/L (SD 1.13), and serum creatinine was 103 µmol/L (SD 96).

The mean total OHAT score was 4.99 (SD 2.02), indicating a moderate oral health burden. Across OHAT domains, most participants were rated as “change” rather than healthy or unhealthy for lips (85.8%), tongue (76.7%), saliva (64.2%), and oral cleanliness (52.5%). For gums and oral tissues, just over half of participants were rated as healthy (52.5%), with only 4.2% rated as unhealthy.

Most participants retained natural teeth (92.4%). Dental decay was present in 52.7% of participants, including 12.7% with four or more decayed teeth. Dentures were present in 21.7% (*n* = 26) of participants; among individuals with dentures, 23.1% had poor denture condition. Oral cleanliness was rated as moderate or poor in 85.0% of participants, and dental pain was reported occasionally or frequently by 11.7%.

Periodontal screening indicated that 71.4% had moderate‐to‐severe periodontal findings (PSR ≥ 3), while 28.6% had no or mild findings (PSR 0–2).

### 3.2 Univariable Associations Between OHAT Domains and Periodontal Screening Findings

Binary logistic regression analyses (Table 2) were based on 109–112 participants, depending on data completeness for each OHAT domain.

**Table 2:** Univariable logistic regression predicting moderate‐to‐severe periodontal screening findings.

Predictor	*N*	OR	95% CI	*p‐*value	Notes
OHAT Lips (Change/Unhealthy vs. Healthy)	112	1.2	0.37–3.63	0.798	Not significant
OHAT Tongue Change/Unhealthy vs. Healthy)	112	3.2	1.30–8.02	0.012	Significant
OHAT Gums & Oral Tissues (Change/Unhealthy vs. Healthy)	112	4.6	1.78–11.85	0.002	Significant
OHAT Saliva (Change/Unhealthy vs. Healthy)	112	2.3	0.99–5.43	0.052	Borderline
OHAT—Decayed teeth (0 = none; 1 = 1–3 teeth; 2 = ≥ 4 teeth)	109	2.1	1.05–4.05	0.034	Significant
OHAT—Oral Cleanliness (Change/Unhealthy vs. Healthy)	112	2.5	0.83–7.65	0.103	Not significant
OHAT—Dental Pain Change/Unhealthy vs. Healthy)	112	1.4	0.35–5.39	0.642	Not significant
Total OHAT burden (> 5 vs. ≤ 5)	112	7.000	2.25–21.76	< 0.001	Significant

*Note:* Outcome was periodontal status (PSR binary: no/mild = 1; moderate‐to‐severe = 1). Each row represents a separate univariable logistic regression model. Models involving natural teeth status and denture presence did not converge due to data separation and are not presented.

Abbreviations: CI, confidence interval; OHAT, Oral Health Assessment Tool; OR, odds ratio; PSR, Periodontal Screening and Recording.

Changes/unhealthy ratings for gums and oral tissues were associated with higher odds of moderate‐to–severe periodontal screening findings (OR 4.59, 95% CI 1.78–11.85, *p* = 0.002). Tongue change/unhealthy ratings were also significantly associated (OR 3.23, 95% CI 1.30–8.02, *p* = 0.012). Saliva change/unhealthy ratings showed a borderline association (OR = 2.33, 95% CI 0.99–5.43, *p* = 0.052).

Increasing categories of decayed teeth were associated with higher odds of moderate‐to‐severe periodontal screening findings (OR 2.06 per category increase, 95% CI 1.05–4.05, *p* = 0.034).

Lip status, oral cleanliness, and dental pain were not significantly associated with the outcome (all *p* > 0.10). Models involving natural teeth status and denture presence did not converge due to data separation and are not presented.

Participants with higher total OHAT scores (> 5 vs. ≤ 5) had significantly higher odds of moderate‐to‐severe periodontal screening findings (OR 7.00, 95% CI 2.25–21.76, *p* < 0.001).

### 3.3 Covariate‐Adjusted Association Between OHAT Domains and Periodontal Screening Findings

Exploratory adjusted analyses (Table 3) were conducted by entering one covariate at a time into separate logistic regression models for each OHAT domain. Associations for tongue, gums, and oral tissues and total OHAT burden remained broadly consistent across models.

**Table 3:** Adjusted logistic regression analyses of OHAT findings and cumulative OHAT burden in relation to moderate‐to‐severe periodontal findings.

OHAT predictor	Adjustment variable	*n* analyzed	Adjusted OR	95% CI	*p*‐value
OHAT—Lips (Change/Unhealthy vs. Healthy)	Age (years)	112	0.91	0.24–3.44	0.890
	Diabetes type (Type 2 vs. Type 1)	112	1.04	0.30–3.60	0.957
	Gender (female vs. male)	112	1.16	0.37–3.60	0.803
	HbA1c (NGSP, %)	108	1.09	0.34–3.51	0.882
	Creatinine (mmol/L)	110	1.09	0.34–3.50	0.890
	Total cholesterol (mmol/L)	104	1.05	0.30–3.64	0.945
	LDL cholesterol (mmol/L)	103	1.04	0.29–3.68	0.948
OHAT—Tongue (Change/Unhealthy vs. Healthy)	Age (years)	112	2.64	0.89–7.83	0.081
	Diabetes type (Type 2 vs. Type 1)	112	2.32	0.87–6.18	0.092
	Gender (Female vs. Male)	112	3.28	1.31–8.20	0.011
	HbA1c (%)	108	3.38	1.32–8.64	0.011
	Creatinine (mmol/L)	110	3.33	1.32–8.39	0.011
	Total cholesterol (mmol/L)	104	3.37	1.30–8.76	0.013
	LDL cholesterol (mmol/L)	103	3.49	1.32–9.25	0.012
OHAT—Gum and oral tissues (Change/Unhealthy vs. Healthy)	Age (years)	112	2.7	0.96–7.64	0.059
	Diabetes type (Type 2 vs. Type 1)	112	4.1	1.53–11.23	0.005
	Gender (Female vs. Male)	112	4.7	1.80–12.10	0.002
	HbA1c (%)	108	5.2	1.90–14.26	0.001
	Creatinine (mmol/L)	110	4.5	1.72–11.65	0.002
	Total cholesterol (mmol/L)	104	5.3	1.90–14.62	0.001
	LDL cholesterol (mmol/L)	103	5.4	1.93–15.06	0.001
OHAT—Saliva (Change/Unhealthy vs. Healthy)	Age (years)	112	1.30	0.48–3.55	0.608
	Diabetes type (Type 2 vs. Type 1)	112	1.94	0.78–4.83	0.154
	Gender (Female vs Male)	112	2.33	0.99–5.45	0.052
	HbA1c (%)	108	2.23	0.94–5.32	0.071
	Creatinine (mmol/L)	110	2.13	0.89–5.08	0.088
	Total cholesterol (mmol/L)	104	1.95	0.79–4.79	0.146
	LDL cholesterol (mmol/L)	103	1.94	0.77–4.85	0.158
OHAT—Decayed teeth (0 = none; 1 = 1–3 teeth; 2 = ≥ 4 teeth)	Age (years)	109	1.74	0.83–3.65	0.142
	Creatinine (mmol/L)	107	2.13	1.08–4.18	0.029
	Diabetes type (Type 2 vs. Type 1)	109	1.69	0.83–3.45	0.152
	Gender (female vs. male)	109	2.06	1.05–4.03	0.035
	HbA1c (NGSP %)	105	1.88	0.93–3.80	0.080
	Creatinine (mmol/L)	107	2.13	1.08–4.18	0.029
	Total cholesterol (mmol/L)	101	2.37	1.13–4.50	0.023
	LDL cholesterol (mmol/L)	100	2.22	1.04–4.71	0.034
Oral cleanliness (Change/Unhealthy vs. Healthy)	Age (years)	112	2.36	0.58–9.58	0.229
	Diabetes type (Type 2 vs. Type 1)	112	2.09	0.63–6.95	0.230
	Gender (female vs. male)	112	2.52	0.83–7.67	0.103
	HbA1c (NGSP %)	108	2.29	0.74–7.08	0.150
	Creatinine (mmol/L)	110	2.55	0.83–7.86	0.103
	Total cholesterol (mmol/L)	104	2.91	0.90–9.40	0.074
	LDL cholesterol (mmol/L)	103	2.92	0.89–9.63	0.077
OHAT—Dental pain (Change/Unhealthy vs. Healthy))	Age (years)	112	1.07	0.23–4.95	0.930
	Diabetes type (Type 2 vs. Type 1)	112	1.25	0.29–5.32	0.767
	Gender (female vs. male)	112	1.39	0.36–5.42	0.638
	HbA1c (NGSP %)	108	1.27	0.32–5.06	0.732
	Creatinine (mmol/L)	110	1.35	0.34–5.33	0.669
	Total cholesterol (mmol/L)	104	1.48	0.37–6.02	0.582
	LDL cholesterol (mmol/L)	103	1.54	0.38–6.30	0.548
Total OHAT score burden (binary)	Age (years)	112	4.65	1.40–15.46	0.012
	Diabetes type (Type 2 vs. Type 1)	112	5.95	1.84–19.30	0.003
	Gender (female vs. male)	112	7.33	2.32–23.11	< 0.001
	HbA1c (NGSP %)	108	6.82	2.12–21.91	0.001
	Creatinine (mmol/L)	110	7.33	2.35–22.90	< 0.001
	Total cholesterol (mmol/L)	104	7.03	2.20–22.47	< 0.001
	LDL cholesterol (mmol/L)	103	6.97	2.17–22.36	0.001

*Note:* Each adjusted model was fitted separately for one covariate due to sample size constraints. Outcome was periodontal status (PSR binary: no/mild = 0; moderate‐to‐severe = 1).

Abbreviations: CI, confidence interval; OHAT, Oral Health Assessment Tool; OR, odds ratio; PSR, Periodontal Screening and Recording.

For tongue, adjusted ORs ranged from 2.32 to 3.49 and were statistically significant in models adjusted for gender, HbA1c, creatinine, total cholesterol, and LDL cholesterol. For gums and oral tissues, adjusted ORs ranged from 2.71 to 5.39 and remained statistically significant in most models, particularly when adjusted for diabetes type, gender, HbA1c, creatinine, total cholesterol, and LDL cholesterol.

Decayed teeth remained significantly associated with moderate‐to‐severe periodontal screening findings in several adjusted models, including those accounting separately for creatinine, gender, total cholesterol, and LDL cholesterol.

Total OHAT burden showed the most consistent association, with adjusted ORs ranging from 4.65 to 7.33 and all corresponding *p*‐values ≤ 0.012. These findings indicate that greater cumulative OHAT‐assessed oral health burden remained associated with moderate‐to‐severe periodontal screening findings after accounting individually for demographic and biomedical covariates.

### 3.4 Screening Performance of OHAT Domains (Table 4)

**Table 4:** Screening performance of OHAT items for identifying PSR‐defined moderate periodontal disease.

OHAT measure	Sensitivity (%)	Specificity (%)	PPV (%)	NPV (%)	*n*
OHAT Lips (0 = Health; 1 = Change/Unhealthy)	86.3	15.6	71.9	31.3	112
OHAT Tongue (0 = Health; 1 = Change/Unhealthy)	82.5	40.6	77.6	48.1	112
OHAT Gum and oral tissue (0 = Health; 1 = Change/Unhealthy)	56.3	78.1	86.5	41.7	112
OHAT Saliva (0 = Health; 1 = Change/Unhealthy)	72.5	46.9	77.3	40.5	112
OHAT Natural teeth (Yes = 0; No = 1)	2.5	100.0	100.0	27.9	111
Oral cleanliness (0 = Health; 1 = Change/Unhealthy)	90.0	21.9	74.2	46.7	112
OHAT Dental pain (0 = Health; 1 = Change/Unhealthy)	12.5	90.6	76.9	29.3	112
OHAT Dentures present (Yes = 1; No = 0)	23.8	100.0	100.0	34.4	112
OHAT Decayed teeth (≥ 1 vs. none)	59.7	62.5	79.3	39.2	109
Total OHAT score burden (> 5 vs. ≤ 5)	50.0	87.5	90.9	41.2	112

*Note:* Sensitivity indicates the percentage of participants with moderate‐to‐severe periodontal screening findings correctly identified (PSR = ≥ 3 in ≥ 1 sextant). Specificity indicates the percentage of participants with no/mild periodontal screening findings correctly excluded (PSR 0–2 in all sextants).

Abbreviations: NPV, negative predictive value; PPV, positive predictive value.

Screening performance varied across OHAT domains. Lip condition and oral cleanliness demonstrated high sensitivity (86.3% and 90.0%, respectively) but low specificity (15.6% and 21.9%, respectively), indicating limited discriminatory ability when used alone.

Tongue, saliva, and gums and oral tissues showed more balanced screening characteristics. Among these, gums and oral tissues demonstrated the highest specificity (78.1%) and PPV (86.5%), although sensitivity was lower (56.3%). Decayed teeth showed moderate sensitivity (59.7%) and specificity (62.5%). Some indicators, such as the absence of natural teeth or denture presence, showed high specificity but poor sensitivity and limited value as stand‐alone screening markers.

The total OHAT score (> 5 vs. ≤ 5) demonstrated higher specificity (87.5%) and positive predictive value (90.9%), but moderate sensitivity (50.0%) and modest negative predictive value (41.2%). These findings suggest that higher OHAT burden may help identify individuals with moderate‐to‐severe periodontal findings, although low scores do not reliably exclude periodontal involvement.

## 4 Discussion

This study examined whether observable oral health changes assessed using the Oral Health Assessment Tool (OHAT) were associated with periodontal screening findings among adults with diabetes attending outpatient clinics. Using the Periodontal Screening and Recording (PSR) system as a screening reference standard, several OHAT domains, particularly gums and oral tissues, tongue condition, and decayed teeth, were associated with moderate‐to‐severe periodontal findings. Among these, the total OHAT burden showed the strongest and most consistent association across analyses.

### 4.1 OHAT and Periodontal Screening Alignment

OHAT was developed to support recognition of observable oral health changes by healthcare professionals without dental training and to prompt referral rather than to establish diagnosis (Chalmers et al. 2005; Everaars et al. 2020; Thapa et al. 2021). Previous investigations have primarily focused on feasibility, reliability, and use in aged care and inpatient settings (Chu et al. 2024; Australian Commission on Safety and Quality in Health Care 2023; AIHW 2009), with limited evidence linking OHAT findings to periodontal screening outcomes.

The present findings extend existing evidence by demonstrating that several observable OHAT findings are associated with periodontal screening results in adults with diabetes, a population at elevated periodontal disease risk. Although OHAT does not directly assess periodontal pocket depth or attachment loss, several domains capture visible manifestations of inflammatory and hygiene‐related processes plausibly linked to periodontal pathology.

Notably, total OHAT burden showed a stronger and more consistent association with periodontal screening findings than most individual domains. In clinical practice, oral disease often reflects the accumulation of multiple oral changes rather than a single isolated sign (WHO 2025; Seitz et al. 2019). A structured observable measure capturing overall oral health burden may therefore provide a more informative indicator of periodontal risk than reliance on individual OHAT domains alone.

### 4.2 Domain‐Specific Findings

Changes in gums and oral tissues showed the strongest and most consistent associations with the outcome. This is clinically plausible, as gingival erythema, swelling, and other visible tissue changes reflect inflammatory processes central to periodontal disease (Goulão et al. 2021).

Tongue changes were also associated with the outcome. While not a direct marker for periodontal destruction, tongue status may reflect broader oral hygiene conditions, biofilm accumulation, or co‐occurring oral pathology relevant to periodontal disease risk (du Toit 2003).

Increasing categories of decayed teeth were associated with higher odds of moderate‐to‐severe periodontal screening findings. Although dental caries and periodontal disease are distinct conditions, they share behavioral and systemic determinants, including oral hygiene patterns and glycemic dysregulation (Desai and Nair 2023; Chapple et al. 2017). In this context, decayed teeth may reflect broader oral disease burden rather than a specific marker for periodontal pathology.

In contrast, lip condition, oral cleanliness, and dental pain were not significantly associated with periodontal screening findings. This suggests that some OHAT domains may be more informative than others when the aim is to identify individuals who warrant further periodontal evaluation (Chalmers et al. 2005).

### 4.3 Screening Characteristics and Clinical Interpretation

Screening performance varied across OHAT domains. Domains such as lip condition and oral cleanliness showed high sensitivity and low specificity, meaning they identified most individuals with moderate‐to‐severe periodontal screening findings but also misclassified many without these findings. In contrast, total OHAT burden and changes in gum and oral tissue showed higher specificity and positive predictive value, indicating better identification of individuals requiring referral when findings were positive.

Sensitivity for total OHAT burden was moderate, and negative predictive values were low across domains. Accordingly, OHAT should not be used to rule out periodontal disease. Rather, its value lies in supporting referral decisions in settings where formal periodontal assessment is not routinely available.

It is also important to interpret predictive values cautiously, as they are influenced by disease prevalence. In this sample, over 70% of participants had moderate‐to‐severe periodontal screening findings, which likely contributed to the relatively high positive predictive values observed.

### 4.4 Clinical and Systems Implications

Within outpatient diabetes settings, periodontal screening is rarely embedded despite well‐established links between glycemic control and periodontal inflammation. OHAT may offer a pragmatic observational approach to support early identification of individuals who may benefit from dental referral, particularly where dental professionals are not integrated into the care team.

Implementation, however, requires careful consideration of workflow, training, and referral pathways. Screening without access to follow‐up dental care may increase recognition without improving outcomes. Future research should therefore examine OHAT implementation in routine diabetes services, including feasibility, inter‐rater reliability when administered by non‐dental clinicians, and its potential impacts on referral uptake and clinical outcomes.

## 5 Strengths and Limitations

Strengths of this study include the use of clinically assessed oral health measures, application of a widely recognized periodontal screening system as a reference standard, and inclusion of a real‐world outpatient population reflective of routine diabetes care. The explicit focus on screening rather than diagnosis aligns with the intended use of OHAT in non‐dental settings. Concurrent administration of OHAT and PSR during a single visit reduced temporal variation between assessments.

Several limitations warrant consideration. The cross‐sectional design precludes assessment of temporal relationships between observable oral health changes and periodontal findings. Recruitment from a single tertiary center may limit generalizability to other healthcare settings or populations.

OHAT and PSR assessments were conducted by the same experienced oral health professional rather than by independent assessors, which is an important limitation of the study. Inter‐rater reliability was therefore not evaluated. Furthermore, although OHAT is designed for use by non‐dental healthcare professionals, its performance when administered by non‐dental clinicians was not evaluated in the present study. Future studies should evaluate OHAT when administered independently by trained non‐dental clinicians, with periodontal screening conducted separately by an oral health professional.

PSR was used as a screening reference standard and does not provide a comprehensive periodontal diagnosis, including measures of clinical attachment loss or radiographic bone levels.

Adjusted analyses were exploratory and based on separate one‐covariate models rather than a fully multivariate approach; therefore, residual confounding cannot be excluded. In addition, small subgroup sizes limited the evaluation of some OHAT domains, particularly denture‐related measures, and resulted in model instability for certain variables.

## 6 Conclusion

This study found that several OHAT domains, particularly gums and oral tissues, tongue condition, decayed teeth, and higher total OHAT burden, were associated with moderate‐to‐severe periodontal screening findings among adults with diabetes attending outpatient clinics.

These findings support further evaluation of OHAT as a pragmatic observational screening and referral aid in settings where formal periodontal assessment is not routinely available. However, OHAT should be considered a supportive screening tool rather than a substitute for comprehensive periodontal assessment. As OHAT was administered by an oral health professional in the present study, its performance when used by non‐dental clinicians remains to be established. Longitudinal and implementation‐focused studies are warranted to evaluate its performance when used by non‐dental clinicians and its potential to improve referral pathways and oral health outcomes within integrated diabetes care.

## Author Contributions

**Grace Wong:** conceptualization, investigation, methodology, data curation, data analysis, validation, visualization, writing‐original draft, writing – review and editing. **Sarah Glastras:** conceptualization, methodology, validation, visualization, writing – review and editing. **Sarah Brennan:** conceptualization, methodology, validation, visualization, writing – review and editing. **Marija Saponja:** conceptualization, investigation, methodology, writing – review and editing. **Anna Cheng:** conceptualization, investigation, methodology, writing – review and editing. **Jami Walk:** conceptualization, methodology, project, administration, writing – review and editing. **Kyle Cheng:** conceptualization, investigation, methodology, project administration, writing – review and editing. **Wenpeng You:** methodology, data curation, software, formal analysis, validation, visualization, writing – review and editing.

## Ethics Statement

Ethics approval for this study was obtained from the Northern Sydney Local Health District Human Research Ethics Committee (2023/ETH02593).

## Consent

All participants provided written informed consent prior to participation.

## Conflicts of Interest

The authors declare no conflicts of interest.

## Data Availability

The data that support the findings of this study are available on request from the corresponding author. The data are not publicly available due to privacy or ethical restrictions.
